# Real-life efficacy and safety of oral propranolol for ocular adnexal infantile hemangiomas: observational cohort study

**DOI:** 10.3389/fopht.2025.1493171

**Published:** 2025-05-05

**Authors:** Stefano Malvindi, Elena Sammarco, Andrea Elefante, Vittoria Lanni, Domenico Cicala, Francesco Esposito, Ciro Picardi, Adriana Iuliano, Dana Cohen, Giuseppe Mariniello, Antonella D’Aponte, Ciro Costagliola, Francesco Briganti, Diego Strianese

**Affiliations:** ^1^ Department of Neurosciences, Reproductive Sciences and Dentistry. University of Naples Federico II, Naples, Italy; ^2^ Pediatric Neuroradiology, Department of Neuroscience, Santobono-Pausilipon Children’s Hospital, Naples, Italy; ^3^ Department of Advanced Biomedical Sciences, University of Naples Federico II, Naples, Italy; ^4^ Division of Neurosugery, Department of Neuroscience, Reproductive and Odontostomatological Sciences, University of Naples Federico II, Naples, Italy

**Keywords:** oral propranolol, infantile hemangiomas, amblyopia, ocular adnexal, vascular tumor

## Abstract

**Objective:**

To assess the effectiveness and safety of oral propranolol for the treatment of ocular adnexal infantile hemangiomas.

**Patients and methods:**

retrospective observational cohort study. Propranolol was administered at an initial oral dose of 1 mg/kg and subsequently increased to 2 mg/kg for 1 year. Outcomes were evaluated by comparing pre- and post-treatment clinical findings, contrast-enhanced ultrasound (CEUS) findings and/or orbital magnetic resonance imaging findings from baseline to 3, 6, 9, 12, 24, and 48 weeks. Regression was graded as follows: satisfactory when 90% and above of the baseline lesion volume and extension decreased, acceptable when 50 to 90%, mediocre when 30 to 50%, poor less than 30%.

**Results:**

Twenty-four patients were included in this study. The mean age at presentation was 4 ± 1 week. Sixteen (71%) patients were females and 7 (29%) were males. The mean follow-up duration was 18 ± 3 months. Therapy was started for of 23/24 patients at 5 weeks old, of 1/24 started at 9 weeks of age. The median age was 5,16 weeks. Sixteen patients (66%) had satisfactory resolution between 3 and 6 weeks, 5 (20%) after 9 weeks, and 3 (12%) after 12 weeks. One patient (5%) had a mediocre response after 24 weeks. One patient withdrew from therapy because of hypoglycemia, which was successfully managed as an outpatient. No significant adverse reactions, such as bradycardia, hypotension, bronchospasm, or congestive heart failure, were detected in this cohort.

**Conclusion:**

This study indicates that the real-life use of oral propranolol for infantile hemangioma yields a high success rate with a lower morbidity than previously reported, particularly when managed by a proficient and multidisciplinary team.

## Introduction

Infantile hemangioma (IH) is the most common vascular tumor in infancy and is characterized by nonlinear proliferation of endothelial cells. It is considered a benign vascular tumor according to the International Society for the Study of Vascular Anomalies (ISSVA).

It occurs in 4-10% of European and Northern American infants, with a lower incidence in African American and Asian counterparts. It is more common in females than males and is more frequently observed in premature infants and those with a low birth weight (1, 2).

Other recognized risk factors include maternal smoking, older maternal age, *in vitro* fertilization, amniocentesis, chorionic villus sampling, maternal bleeding during the first trimester, placenta previa, and pre-eclampsia (3–6).

IH typically appears in the first few weeks of life, with the first phase characterized by a rapid proliferation of endothelial cells that last 5–8 weeks. At this time, they generally reach 80% of their size, followed by a slower growth phase until 9–12 months of age. A longer proliferative phase is more common in large, segmental, and deep IH and in IH located in the head and neck region, including the ocular adnexa (such as the periocular and orbital areas) and the parotid region. The subsequent involutional phase starts at around 12 months of age and usually lasts for 3 to 9 years, leaving residual change in up to 69% of the patients (7–10). This lesion may present as a small isolated lesion or as a large mass with visual impairment that can occur in any area of the skin, but most commonly in the ocular adnexa region. IH is classified according to the depth of involvement as superficial, deep, or mixed. The clinical presentation of IH depends on the depth of the lesion. Superficial IHs are located in the superficial dermis and present as red and finely lobulated plaques. Deep IHs occupy the deep dermis and/or subcutaneous tissue and are present as skin-colored or blue subcutaneous masses. Mixed IHs involves both layers of the dermis and, often, the subcutis, and exhibits clinical features of both superficial and deep IH (11).

The diagnosis of Infantile Hemangioma is generally based on clinical features; in selected cases, MRI and ultrasound could allow differential diagnosis and assist in defining the size and deep extension of the lesion.

Biopsy may be needed for the differential diagnosis of malignancy, particularly mesenchymal tumors, such as rhabdomyosarcoma (12, 13).

Although most IHs cases have a favorable natural history, up to 10% can lead to significant complications, particularly when located in the ocular adnexa region, such as astigmatism (33%), visual axis obstruction (29%), nasolacrimal duct obstruction (7%), ptosis (4%), amblyopia (3%), and strabismus (1%) (14, 15).

Cosmetic sequelae are significant in approximately half of untreated IH cases and include telangiectasia fibrofatty tissue. anetoderma, redundant skin, and scarring (16, 17).

Several treatment options are available for IH, including observation, local or oral steroids, interferon, local and oral beta-blockers, laser therapy, and surgery. IH likely to require treatment should be treated in a proper timeframe, ideally by 5 weeks of age (18). indeed, prompt treatment is required for those cases threatening visual function to any extent, such as ptosis with severe limitation of the visual field and risk of amblyopia and large lesions compressing the eye. The choice of therapy depends on the location, size, and associated complications of the lesions. Oral propranolol is currently considered the preferred therapy for complicated IH and has been shown to be effective in reducing the size and associated complications of IH. Topical and intralesional timolol are also effective for the treatment of small superficial IH. Laser therapy is useful for the treatment of residual telangiectasias after involution. Surgical excision is reserved for cases that do not respond to the aforementioned therapies, or for those presenting with significant disfigurement or functional impairment (19). Herein, we report the outcomes of a cohort of patients with ocular adnexal Infantile Hemangioma treated with oral propranolol.

## Materials and methods

This was a retrospective cohort study that used data from the patients’ medical records. This study was approved by the Ethics Committee of the Children’s Hospital.

Patients with a confirmed diagnosis of ocular adnexal IH were included in the study.

The diagnosis was based on the accepted clinical and radiological findings (14, 15). The exclusion criteria were allergies to ingredients, bradycardia, hypotension, and previous heart block.

Charts of twenty-four children with periocular and orbital IH referred to Pediatric department of the ‘Santobono’ Pediatric Hospital in Naples between January 2011 and December 2021 were reviewed.

Clinical datas such as patient demographics, age, duration of signs and symptoms, clinical presentation, location of the IH, imaging findings, type of treatment, complications, and subsequent follow-up were collected. All patient data were deidentified.

All patients underwent a complete ophthalmic examination, cardiac evaluation to rule out pre-existing disorders, allergies tests for asthma, blood tests for glycemia, and measurement of blood pressure.

Periocular and orbital extension of the lesions were assessed using three methods: clinical evaluation of volume and extension of the lesion objectively registered using a ruler and photographic evaluation; evaluation of capillary density using contrast-enhanced ultrasound (CEUS); and evaluation of volume and extension using magnetic resonance imaging (MRI).

Patients underwent MRI to assess the extent of intraorbital lesions and additional maxilofacial segmental components.

Our MRI protocol included thin-slice (2.5 mm) scans in the axial and coronal planes with TSE T1, T2 SPIR with fat suppression, DWI and 3D TFE T1 Fat suppressed after contrast injection with a 1.5T MRI device (Ingenia, Philips Medical Systems); sometimes dynamic sequences and MRA were added. Tumor size and volume were calculated in cm3 by experienced neuroradiologists with manual segmentation from pre- and postoperative volumetric TFE scans using commercially available software (HorosTM 4.0.1). Segmentation could include the hemangioma-infiltrated lacrimal lodge in the supero-external orbital portion, computed in both pre- and post-treatment images. Furthermore, the degree of exophthalmos was evaluated using the Cabanis oculo-orbital index (percentage of the anteroposterior axis of the eyeball located beyond the interzygomatic line on the axial MRI plane; normal value <70%); it distinguishes exophthalmos of grade I (70–100%), grade II (equal to 100%) and grade III (over 100%). The MRI pattern was typically that of a well-circumscribed lesion, hypointense to the extraocular muscle on T1-weighted sequence, markedly hyperintense on T2-weighted sequence with intense contrast enhancement. Volume changes, relationships with orbital structures and residual lesion components after treatment were evaluated.

The treatment protocol included propranolol at an initial oral dose of 1 mg/kg in two daily doses for 7 days. If no side effects occurred, the dose was increased to 2 mg/kg twice daily for one year.

Parents were informed about potential side effects such as hypotension, cold extremities, bronchial hyperreactivity, and hypoglycemia, and were educated to recognize and manage them early.

CEUS and MRI were performed for every patient at baseline, then repeated at three months and one year of therapy, respectively, to assess the progress of lesion regression and monitor any changes in size, volume, and vascular characteristics over time.

The volume of the lesions was measured using imaging techniques, particularly MRI, which allowed for precise and objective volume assessment through manual segmentation of pre- and post-treatment scans.

CEUS and MRI were repeated after three months and one year of therapy, respectively. Glycaemia and blood pressure after 2 hours of treatment.

Close daily follow-up was initially performed for 3 weeks to evaluate potential side effects, after which patients continued therapy at home and returned to the hospital once a month for follow-up. Treatment was continued during the lesion regression phase. After one year, if there was a complete regression, the therapy was interrupted; otherwise, the dosage of the drug was increased to 3 mg/kg in three doses up to 18 months. The therapeutic endpoint was graded as follow: satisfactory when the baseline lesion volume and extension decreased 90% and above, acceptable from 50 to 90%, mediocre from 30 to 50%, poor less than 30%. Other treatment endpoints were assessed on CEUS and MRI, particularly evaluating vascular density, as follows: sparse vascular density was considered matching satisfactory effect; a slightly richer vascular density was considered matching with acceptable; still rich vascular density was considered disappointed treatment outcome matching with mediocre and poor, although the blood vessel density was lower than the initial.

## Results

The Twenty-four patients with periocular and orbital IH, whose details are reported in **Table 1**, were included in this cohort. The mean age at presentation was 4 ± 1 weeks, and 8 were preterm babies. Of the 24 patients, 17 were female (71%) and 7 were male (29%). The average follow-up time was 18 ± 3 months. Therapy was started for 23/24 patients at 5 weeks old 1/24 started at 9 weeks old. Thirteen (54%) patients had right ocular adnexal area involvement and 11(46%) had left-side involvement. None of the patients had bilateral disease. The most common presenting symptom was eyelid swelling, which was observed in 19 (79%) patients. Other clinical signs included eyelid telangiectasia in 10 patients (42%), proptosis in 8 patients (33%), and strabismus in 1 patient (4%). Four infants had PHACE syndrome with extraocular findings. Twenty patients showed a predominant strawberry pattern. Fifteen patients had a combination of orbital involvement and a large subcutaneous extension.

**Table 1:** Case series.

Patient n.	Sex	Patient Age (months) at Treatment Onset (mos)	Lesion Progression	Risk of Amblyopia	Treatment Duration	Response to Treatment	Type of Hemangioma	Major Side Effect
1	F	5	worsening	Astigmatism	12	after 4 weeks	Deep	NO
2	F	5	worsening	Occlusion	12	after 9 weeks	Deep	NO
3	F	5	worsening	Ptosis	12	after 4 weeks	Mixed	NO
4	F	5	worsening	Occlusion	12	after 15–20 weeks	Mixed	NO
5	F	5	stable	Occlusion	12	after 4 weeks	Mixed	NO
6	M	5	worsening	not observed	12	after 4 weeks	Mixed	NO
7	F	5	worsening	Occlusion	12	after 9 weeks	Deep	NO
8	F	5	worsening	Astigmatism	12	after 4 weeks	Mixed	NO
9	F	5	worsening	not observed	12	after 4 weeks	Mixed	NO
10	M	5	stable	Astigmatism	12	after 15–20 weeks	Deep	NO
11	F	5	worsening	not observed	12	after 4 weeks	Deep	NO
12	F	5	worsening	Occlusion	12	after 9 weeks	Mixed	NO
13	M	5	worsening	not observed	12	after 4 weeks	Mixed	NO
14	F	5	worsening	not observed	12	after 4 weeks	Deep	NO
15	F	5	worsening	Occlusion	12	after 4 weeks	Mixed	NO
16	M	9	worsening	Ptosis	18	after 15–20 weeks	Deep	NO
17	M	5	stable	Occlusion	12	after 4 weeks	Mixed	NO
18	F	5	worsening	not observed	12	after 9 weeks	Mixed	NO
19	F	5	worsening	not observed	12	after 4 weeks	Deep	NO
20	F	5	worsening	not observed	12	after 4 weeks	Mixed	NO
21	M	5	stable	not observed	12	after 4 weeks	Deep	NO
22	M	5	worsening	Occlusion	12	after 4 weeks	Mixed	NO
23	F	5	worsening	not observed	12	after 9 weeks	Mixed	NO
24	F	5	worsening	not observed	12	after 4 weeks	Mixed	NO

1. Summary of Patient Characteristics affected by Ocular Adnexal Infantile Hemangioma.

Lesion features on CEUS were hyper-enhancement in the early arterial phase, iso-enhancement in the venous phase in 22 cases, iso-enhancement in the early phase, 1 hyper-enhancing in the late phase in one patient.

MRI pattern was a well-circumscribed lesion hypointense to the extraocular muscle on T1 sequence, hyperintense on T2 sequence, and hyperintense with contrast enhancement in all patients.

The median age at treatment was 5,16 weeks old. The oldest patient was 9-weeks old.

**Figure 1:**
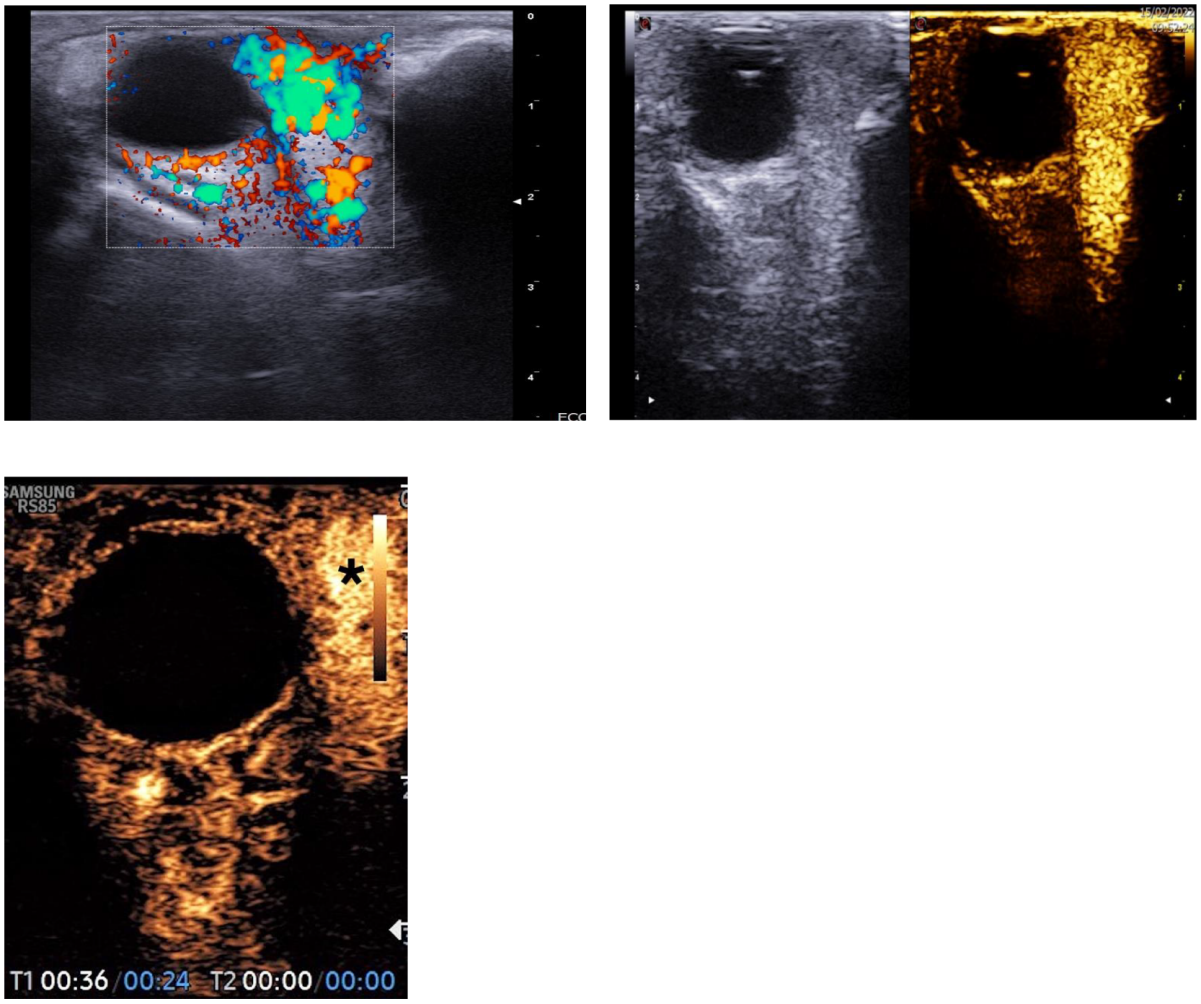
Uniform impregnation of the infantile hemangioma of the orbit (*), prior to drug treatment.

**Figure 2:**
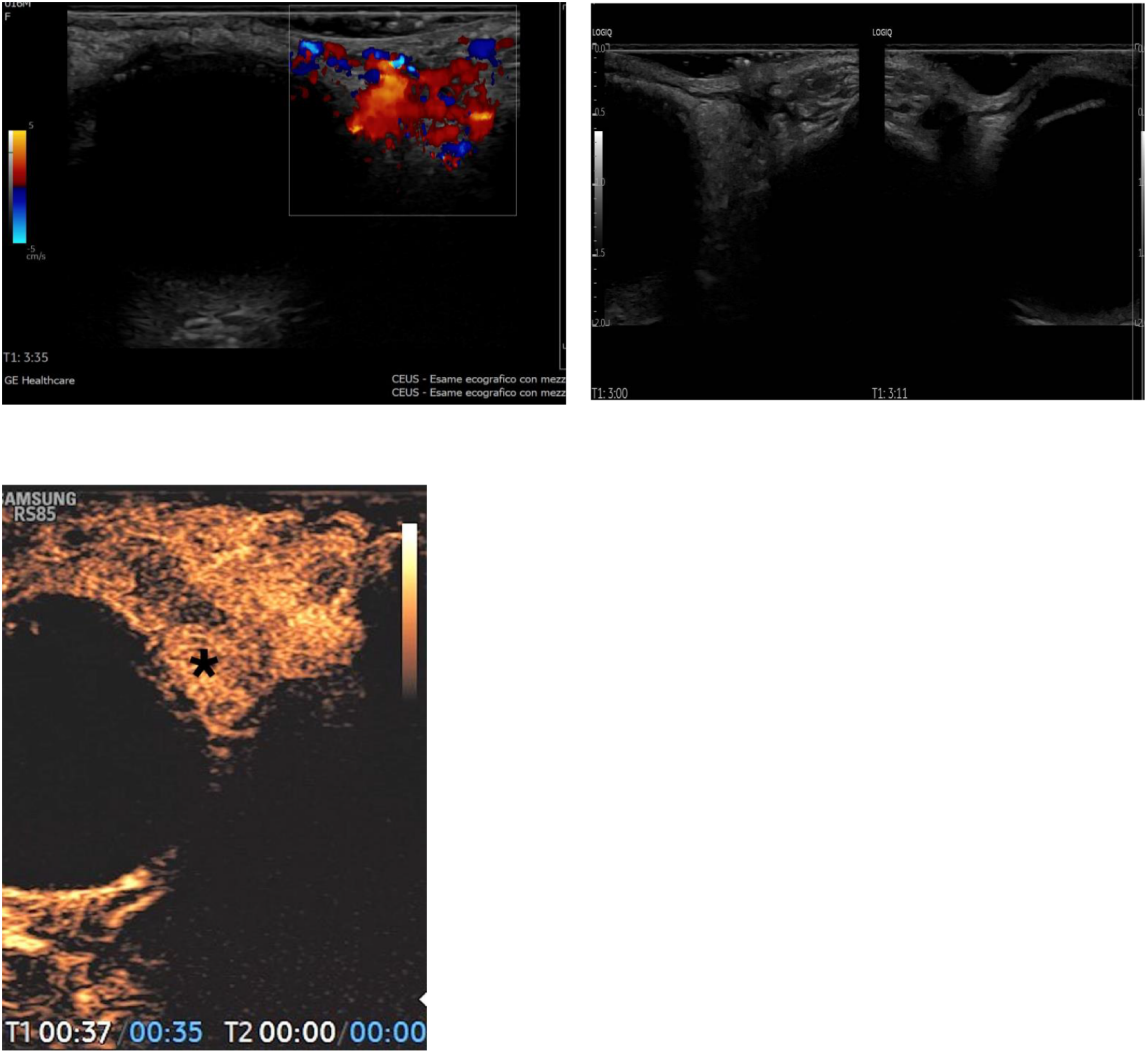
Reduction in volume and intensity of the enhancement of the infantile hemangioma of the orbit after therapy.

**Figure 3:**
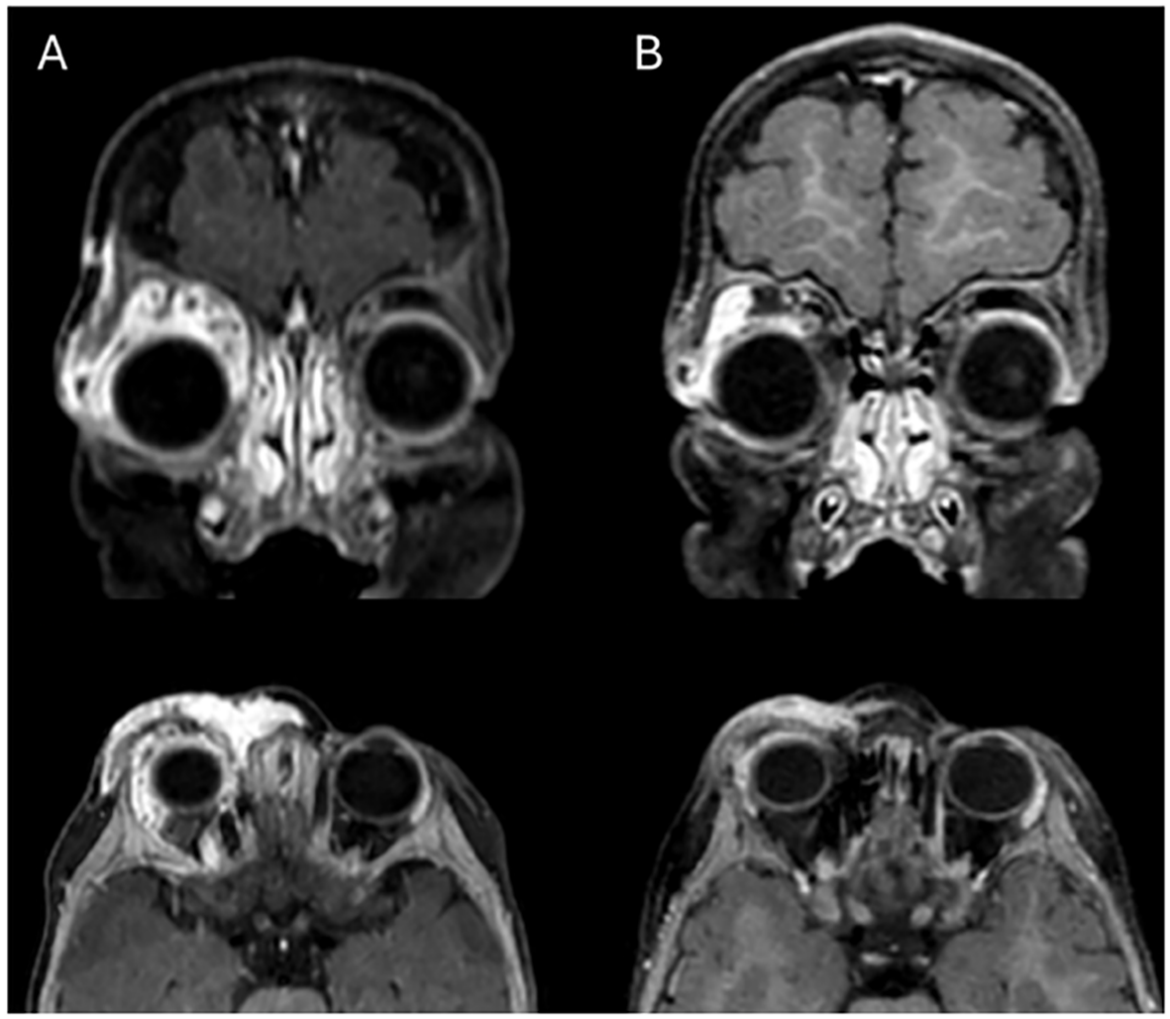
Coronal and Axial contrast enhanced FSPGR T1 MR Images before **(A)** and after **(B)** the treatment. Significant volumetric reduction of the right periorbital hemangioma is observed; note the residual component in the supero-external orbital side; slight reduction of compression on the eyeball.

**Figure 4:**
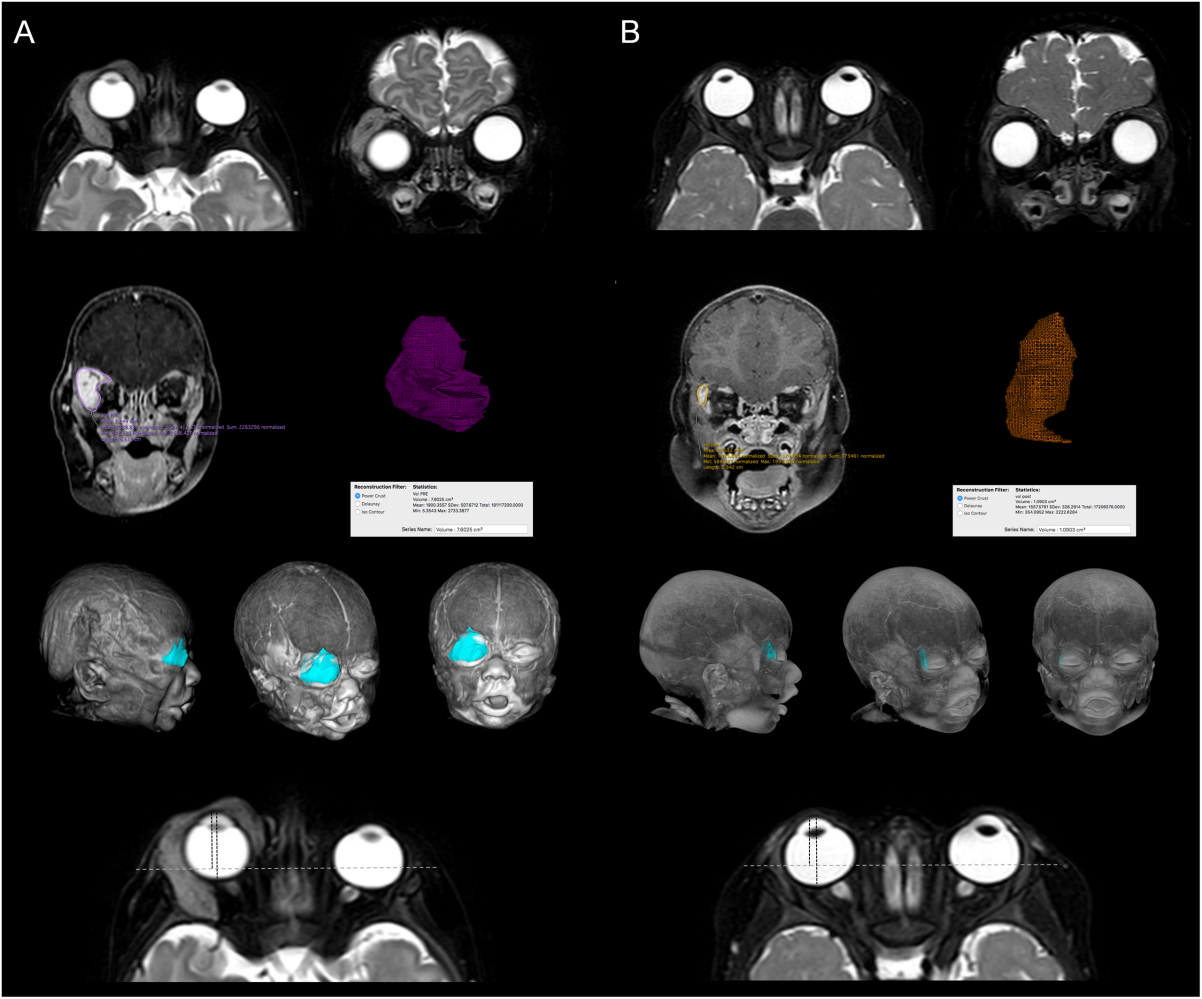
Comparison of MRI images before **(A)** and after treatment **(B)**. Axial and Coronal T2 SPIR MR Images show almost complete resolution of the right periorbital hemangioma; note the resolution of the proptosis due to the reduction of the compression of the eyeball. Segmentation, volume calculation and 3D representation of the periorbital hemangioma and the residual portion after treatment are showed. Resolution of exophthalmos is also detected (Cabanis oculo-orbital index 84% vs 69%).

After 12 months in 95% of patients we had a regression of the mass (**Figures 1**–**4**).

No patients in our cohort developed amblyopia during the study period: the successful reduction of the mass in all patients contributed to the preservation of normal visual development. In one case (5%), we had only a 40% mass reduction after 1 year of treatment, so we improved to 3 mg/kg until 18 months of age and reached a 60% mass reduction, and we stopped the treatment.

Sixteen patients (70%) had a rapid response to treatment after 4 weeks, 5 (20%) after 9 weeks, and 10% (2) showed improvement after 15–20 weeks.

All causes of high risk of amblyopia were resolved after treatment because of the reduction in the mass; no patient required ptosis and strabismus surgical repair.

One patient had hypoglycemia, which was managed as an outpatient.

## Discussion

IH is the most common vascular tumor in infants, affecting 8-10% of the pediatric population, with 80% of cases localized in the head and neck region (20, 21).

The International Society for the Study of Vascular Anomalies (ISSVA) classification system categorizes vascular lesions into two primary groups: tumors, which are true proliferative neoplasms, and malformations, which comprise lesions with defects in morphogenesis. This classification system has significantly impacted the diagnosis and management of these lesions when they involve the ocular-adnexal area. If left untreated, visual sequelae are likely to occur, with amblyopia being a primary concern due to stimulus deprivation, induced anisometropia, secondary strabismus, or globe displacement (22–31). Refractive/anisometropic amblyopia is the most common visual complication associated with periocular hemangiomas, followed by deprivational amblyopia. While the former may be managed with optical correction alone, deprivational amblyopia—caused by obstruction of the visual axis—necessitates early treatment, often during the neonatal period, to prevent irreversible vision loss (32).

Predisposing factors for IH include female sex, low birth weight, and pre-term birth (7). In this vascular tumor, primitive stem cells differentiate into endothelial cells and pericytes (12, 13, 16, 18, 19, 33). Tumor growth occurs in different phases in a nonlinear process. The proliferative phase is characterized by rapid cell proliferation that continues for several months after birth. During this phase, the endothelium grows chaotically and vessels cannot be detected histologically. In the later stage of the proliferation phase, differentiation begins with the formation of enlarged vessels. The involutional phase is characterized by apoptosis of endothelial cells and subsequent deposition of fibro-fatty tissue (18).

IH is described according to color and shape (strawberry nevus, bluish discoloration) (34), and the level of involvement (subcutaneous, deep orbital, combined, segmental, focal, and multifocal) (35). In the present series, 20 patients had a predominant strawberry pattern. All patients included in this study had a deep orbital component; however, 15 patients were considered to have a large subcutaneous extension.

Many IHs are diagnosed through clinical examination; however, imaging can be crucial for deep types with anatomical disfigurement and lesions involving the orbit. Imaging is also useful for assessing the depth of lesions and their relationship with the adjacent structures.

Some studies have found that patients with IH carry mutations in VEGFR1, VEGFR2, or TEM8, indicating that dysregulation of VEGFR1 and VEGFR2 expression may contribute to hemangioma formation (36). Oral propranolol may inhibit angiogenesis by down-regulating the expression of vascular endothelial growth factor (VEGF) in hemangioma-derived stem cells (37, 38).

Several studies have highlighted the efficacy and safety of propranolol in managing IH, especially in cases where traditional treatments like corticosteroids have shown limitations (33, 39, 40). Propranolol has been found to be particularly effective in treating proliferative hemangiomas, with minimal side effects and a high level of safety (41). It is also important to consider that propranolol’s therapeutic efficacy is greatest during the proliferative phase of the hemangioma. When initiated during the involutional stage, clinical response may be less pronounced, underscoring the importance of timely diagnosis and management. Compared to other medications such as nadolol, propranolol has been associated with a lower risk of adverse events, emphasizing its favorable safety profile (34). Although propranolol has demonstrated significant efficacy in treating IH, concerns have been raised regarding its potential impact on pediatric growth and development (35). The use of propranolol has been supported by randomized controlled trials, establishing it as a first-line therapy for IH (33). In clinical practice, the administration of oral propranolol is well tolerated, with few reported complications (42).

In this cohort, oral propranolol was initiated at a dose of 1 mg/kg for a period of one week, with the possibility of increasing the dosage to 2 mg/kg should no adverse effects arise. It is noteworthy that more than 90% of patients demonstrated a satisfactory response within a three-month timeframe. This high rate of efficacy is particularly significant considering that hemangiomas can rapidly grow and obstruct vision if left untreated. In this cohort, the average age at which treatment commenced was five weeks, which may have contributed to the high rate of regression observed. A relevant caveat is that the earlier the treatment is initiated, the better the response. Recent evidence and ISSVA guidelines suggest that propranolol titration from 1 to 3 mg/kg/day can be safely achieved within a week under proper monitoring, allowing for earlier therapeutic dosing. While our protocol adopted a more gradual approach, reaching full dosage at a later stage (around 12–18 months of age), this was mainly due to a highly cautious approach based on the retrospective nature of the study and patient variability. It is also worth noting that, unless in premature infants, the 3 mg/kg/day dosage can be administered in two daily doses, with no need for three administrations. These aspects, along with the timing for discontinuation, should be tailored to the individual patient and ideally align with the structured protocols described in recent literature.

Additional findings of this study revealed no significant adverse effects associated with propranolol, such as bradycardia, hypotension, fatigue, bronchospasm, congestive heart failure, or gastrointestinal discomfort (42). Only one case of hypoglycemia has been reported. This suggests that medical surveillance provided by a highly specialized pediatric hospital may have contributed to the low incidence of complications. Furthermore, previous studies have emphasized the importance of cardiac screening in infants with hemangiomas before starting propranolol treatment to ensure patient safety. The outcomes of this study, which exhibited a high success rate and low morbidity, can be attributed to the implementation of standardized protocols and continuous monitoring. This collaborative care model facilitates prompt identification and management of any potential adverse effects, with the aim of enhancing therapeutic results. A collective body of evidence supports the effectiveness and safety of oral propranolol in addressing ocular adnexal infantile hemangiomas. Future investigations should concentrate on long-term safety and efficacy, particularly by examining the potential impact of propranolol on the growth and development of children.

## Conclusion

This study validated the real-life efficacy of oral propranolol in the treatment of ocular adnexal infantile hemangiomas, as evidenced by the positive response observed in over 90% of patients within three months. The minimal occurrence of adverse effects in conjunction with the benefits of a multidisciplinary approach emphasizes the feasibility of propranolol as a beneficial, effective, and safe option for managing this particular type of hemangioma, providing both practical and aesthetic advantages.

## Data Availability

The raw data supporting the conclusions of this article will be made available by the authors, without undue reservation.
